# IgG4-Related Retroperitoneal Fibrosis in a Patient With B-Cell Lymphoproliferative Disorder

**DOI:** 10.7759/cureus.65118

**Published:** 2024-07-22

**Authors:** Rida Inam, Asim Mehmood, Attia Mahmood, Maaz B Badshah, Usama Shafiq

**Affiliations:** 1 Internal Medicine, Shifa College of Medicine, Islamabad, PAK; 2 Medicine, Ayub Teaching Hospital, Abbottabad, PAK; 3 Gastroenterology and Hepatology, Shifa International Hospitals, Islamabad, PAK; 4 Radiology, Shifa International Hospitals, Islamabad, PAK

**Keywords:** oral steroids, endoscopic ultrasound-guided biopsy, ormond's disease, b-cell lymphoproliferative disorder, retroperitoneal fibrosis

## Abstract

We report an interesting case of a 25-year-old male patient who presented with a complaint of pain in the abdomen for six months, which was not associated with any other symptom, the patient was diagnosed with IgG4-related retroperitoneal fibrosis (RF) via endoscopic ultrasound (EUS)-guided biopsy. He was prescribed steroids and proton pump inhibitors. Due to the limited presentation and rarity of RF, diagnosis of this disease requires extensive diligence and care. In this case report, we underscore the importance of considering the differential diagnosis of RF or Ormond's disease when a patient presents with vague symptoms of pain in the abdomen. According to our knowledge, this is the first case of IgG4-related RF in a patient with B-cell lymphoproliferative disorder reported from Pakistan.

## Introduction

First described by Albarran in 1905 and then detailed by Ormond in 1948, retroperitoneal fibrosis (RF) or Ormond’s disease is characterized by extensive growth of inflammatory fibrotic tissue in the retroperitoneum. It mostly includes the aorta, inferior vena cava, iliac vessels, and ureters. Atypical areas of RF are seen in the perinephric, peripancreatic, and periduodenal areas, and in the pelvis [1]. RF has an expected frequency of around 1.38 per 100,000 patients [2]. Its occurrence in men is marginally higher than that in women, with proportions going from 1:1 to 3:1 [2]. A couple of cases are related to specific causes, for example, neoplasms (lymphomas, sarcomas) or metastatic tumors in the retroperitoneal space), radiotherapy, disease, surgery, trauma, and medications (ergotamine, methyldopa, some beta-blockers, bromocriptine) [1]. Autoimmune etiology for RF is considered because of the relationship of the condition with immune system problems, for example, Hashimoto's thyroiditis, rheumatoid arthritis, lupus erythematosus, Wegener's granulomatosis and autoimmune pancreatitis [1,2]. Our patient did not have any of these mentioned causes or diseases. 

IgG4-related clinically diverse disease can be a cause in approximately half of idiopathic retroperitoneal fibrosis (IRF) cases, according to recent reports [3]. IgG4-related disease (IgG4-RD) is immune-mediated chronic inflammation characterized by enlarged organs, elevated IgG4 levels in the blood, and typical dense lymphoplasmacytic infiltration with enriched in storiform fibrosis, obliterative phlebitis, and IgG4-positive plasma cells. The pancreas, kidney, and retroperitoneum are frequently impacted [4]. Extraordinary fibrosis goes with the inflammatory reaction, prompting super durable organ harm and inadequacy. Demise from IgG4-RD is uncommon [5]. IgG4-RD is a condition that can affect multiple organs, with a particular predilection for the pancreas and biliary tract. Despite its relapsing and remitting course, patients have a good prognosis [5].

## Case presentation

A 25-year-old male, with a known case of B-cell lymphoproliferative disorder, presented to the gastroenterology outpatient department with a complaint of mild pain in the abdomen for the last six months. The pain was localized in the epigastrium and was gradual in onset, but the intensity of the pain worsened with each passing day; dull in character and radiating to the back, the patient gave it a score of eight out of 10 on a pain scale. Neither aggravating nor relieving factors were noted by the patient. Moreover, it was not associated with weight loss, nausea, vomiting, melena, or change in bowel habits. He was also under treatment in Iran for this condition as well as B-cell lymphoproliferative disorder in the peripancreatic region, which was diagnosed in 2023 via positron emission tomography (PET) scan that showed lobulated fluorodeoxyglucose uptake positive avid mass lesion just below the origin of inferior mesenteric vessel extending into the mesenteric region. He also got an endoscopic ultrasound (EUS)-guided biopsy done there, which revealed CD20-positive cells and was also consistent with B-cell lymphoproliferative disorder. The patient’s family history was negative for any type of cancer.

**Figure 1:**
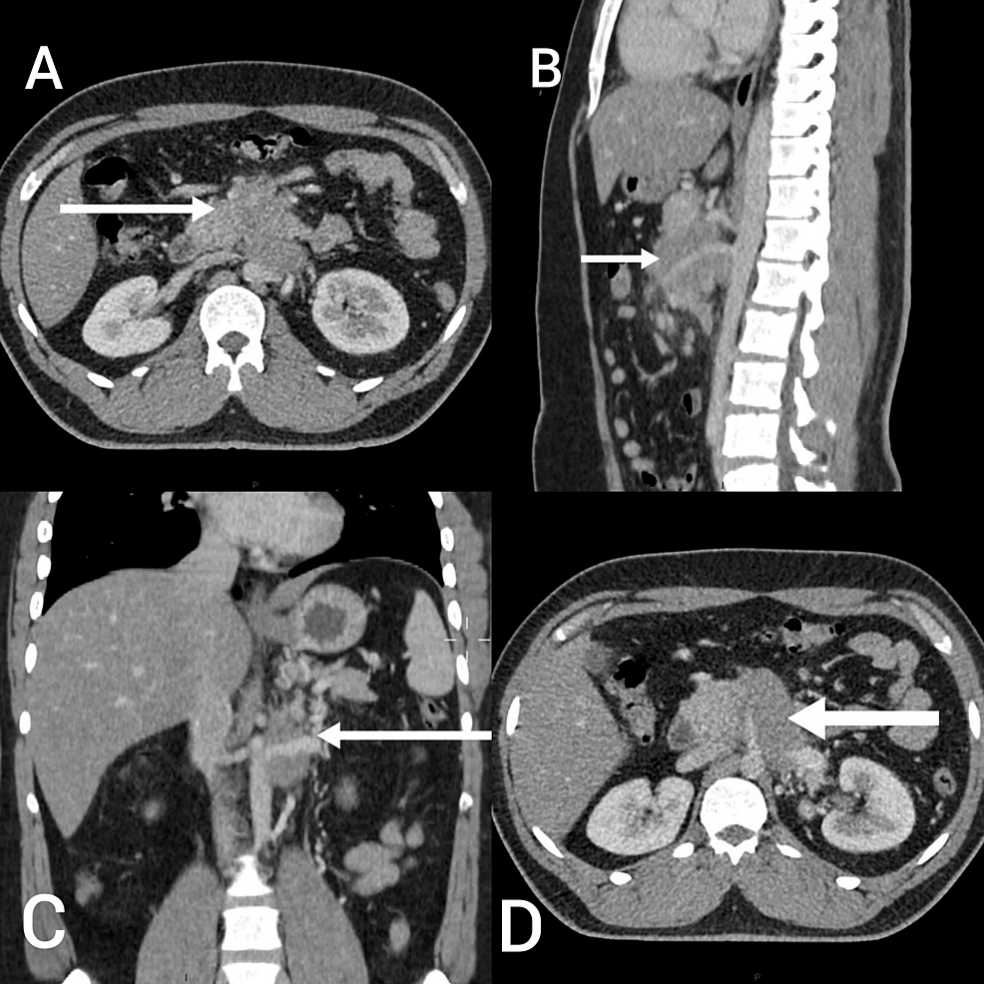
CT scan showing heterogeneously enhancing soft tissue density mass in retroperitoneum. Axial views showing the mass abutting the superior mesenteric vein anterolaterally (A) and inferior vena cava posteriorly (D). Lateral view showing the mass encasing the superior mesenteric artery, loss of fat planes, and inferior vena cava posteriorly (B). Frontal view showing the mass encasing the left renal vein (C).

On examination, the abdomen was soft and non-tender on superficial and deep palpation. No mass was palpable, shifting dullness and fluid thrill were negative, and on auscultation, normal bowel sounds were heard in all four quadrants. Investigations were requested, which included complete blood count, renal and liver function tests, bilirubin, alkaline phosphatase, lactate dehydrogenase, and gamma-glutamyl transferase (GT). Results of all investigations were in the normal range except for alanine transaminase (ALT), which was raised to 60 (normal range: up to 50 U/L). CT scan of the chest, abdomen, and pelvis revealed an ill-defined minimally enhancing hypodense mass in the retroperitoneum inseparable from the pancreatic body inferiorly, encasing major vessels with their resultant narrowing. Misty mesentery with multiple nodes/nodules suggested mesenteric panniculitis (Figure 1).

**Figure 2:**
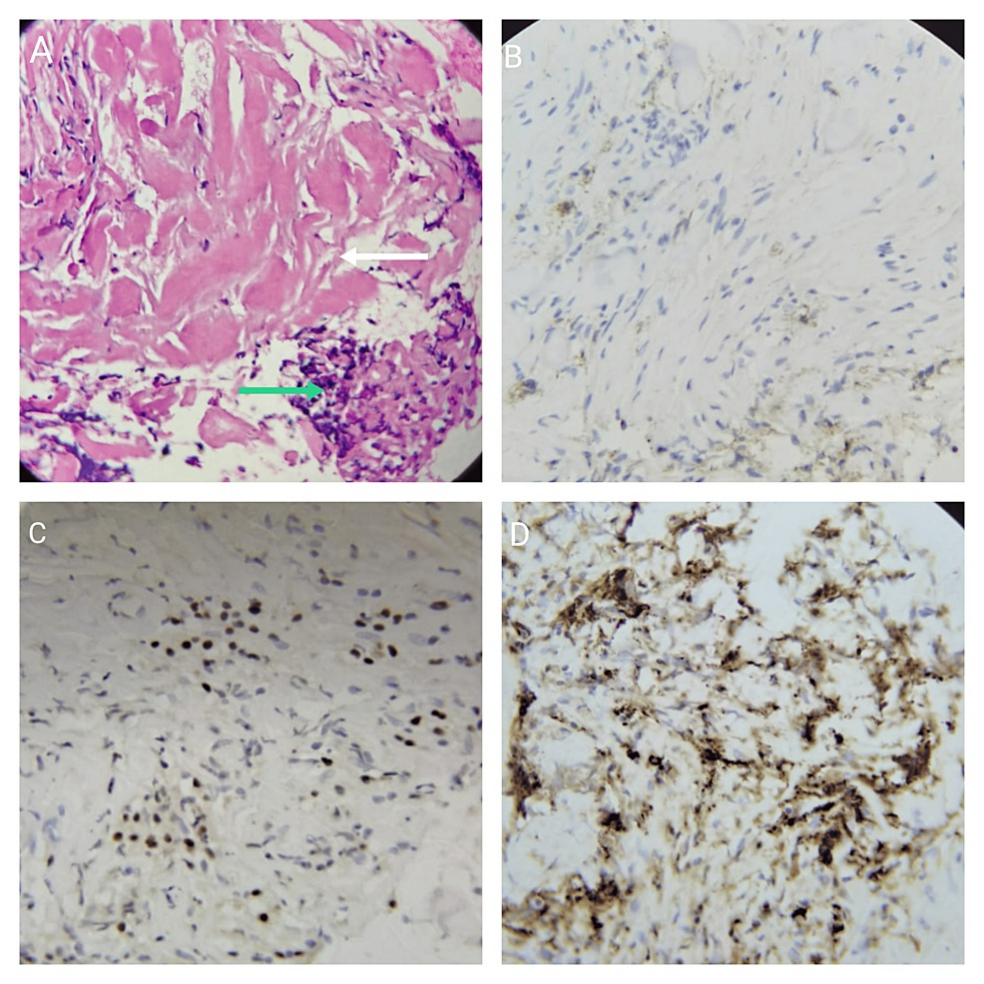
(A) EUS-guided biopsy of the pancreatic mass showing fragments of benign biliary epithelium with dense hyalinization (white arrow) and plasma cells (green arrow) (H&E, 20x obj). Immunohistochemistry CD138 (B) and immunohistochemistry MUM-1 (C) showing increased plasma cells. Immunohistochemistry highlighting IgG4-positive plasma cells (D). EUS: endoscopic ultrasound

Differential diagnosis of pancreatic cancer, tuberculosis, and lymphoma were made. To make the exact diagnosis, an EUS-guided biopsy of the pancreatic mass was obtained, which showed fragments of benign biliary epithelium with mild lymphoplasmacytic infiltrate (containing plasma cells), dense fibrosis, and background hemorrhage. The findings of the biopsy were consistent with IgG4-related RF. On immunohistochemistry, IgG4, CD138, and MUM-1 highlighted plasma cells with only a few cells positive for CD20 (Figure 2).

For the management of the disease, the patient was prescribed deltacortil (40 mg), pantoprazole (40 mg), and multivitamins daily for three months. The patient has been compliant with the medicine and is tolerating it well, without any adverse effects. The patient was told about the advised follow-up after one month with a CT scan of the abdomen and was informed about red flags and to return to the hospital in case of fever, severe pain, and vomiting.

## Discussion

RF grows guilefully because the underlying symptoms are vague. The most widely recognized reports incorporate low back, flank, or abdominal pain, frequently emanating to the groin and additionally to the side of the thigh. The pain aggravation is portrayed as dull and tenacious, with no relief at rest. At first, help is brought by the utilization of non-steroidal anti-inflammatory drugs, however, this treatment is temporary. Less regularly, detailed complications connected with the pressure of retroperitoneally found lymphatic vessels and veins incorporate expanding and profound vein apoplexy of the lower appendages, scrotal enlargement, testicular pain, varicocele, and hydrocele. Constitutional symptoms include muscle and joint pain, fatigue, fever, weight loss, and a lack of appetite. Physical assessment is unremarkable, as in our case. Some of the time abdominal or spine tenderness is available, and seldom a mass can be felt through the stomach wall. The vague clinical picture frequently essentially drags out the time between the beginning of the presentation and the right conclusion, which brings about complexities connected with the high-level fibrotic process [3]. A sign of IgG4-RD is a higher concentration of IgG4 in the blood. The majority of IgG4-RD patients have a serum IgG4 concentration of more than 1350 mg/L, which may decrease during or after treatment with glucocorticoids. For the most part, the degree of serum IgG4 fixation emphatically connects with the number of organs involved. However, raised serum IgG4 alone can't be utilized to analyze or preclude IgG4-RD. Studies reveal that somewhere in the range of 3% and 30% of IgG4-RD patients have normal serum IgG4 fixations [6]. 

Abdominal CT and MRI are the diagnostic radiological techniques used most frequently. CT scan exhibit the size, position, and scope of pathology as well as the impacted region and is useful for follow-up and evaluating the impact of glucocorticoid treatment. Notwithstanding, CT cannot recognize the dynamic sores from fibrosis. The retroperitoneal soft tissue can be easily seen on an MRI. Therefore, MRI is a good alternative as compared to contrast-enhanced CT for patients who have renal insufficiency. Fluorine-18-marked fluorodeoxyglucose (F18 FDG)-PET could identify active disease, and it is of extraordinary importance in tracking down sores in the retroperitoneum and different organs. Subsequently, F18 FDG-PET can be utilized to survey the action and scope of lesions and assess remedial reactions as well as track down residual injuries, yet it cannot recognize malignancies [7]. In IgG4-related RF, the microscopic evaluation is the same as those of IRF; IgG4-related RF shows all the more regularly obliterative phlebitis, a gentle-to-direct eosinophil penetration, and fibrosis with a storiform design. As in IRF, the inflammatory infiltrate is made out of T and B lymphocytes. Even though IgG4-bearing plasma cells might be additionally found in the infiltrate of IRF, what is fundamental for the conclusion of IgG4-RD is a proportion of IgG4-bearing plasma cells to add up to IgG-bearing plasma cells higher than 30-50 % [8]. In our case, histopathology revealed numerous IgG4-positive plasma cells. Glucocorticoid therapy is the first-line treatment, typically starting with 0.6-1 mg/kg/day for two months; then, at that point, the measurements are slowly tightened and kept up with 2.5-5 mg/day for more than six months. In IgG4-RD, the serum IgG4 level is useful to control the dose of glucocorticoids. Maintenance of glucocorticoids frequently cannot be ended to keep away from relapse [9]. 

## Conclusions

RF is an uncommon, rarely reported disorder whose etiology still remains unknown. The feature that makes this case report strikingly interesting is that it presents the case of IgG4-positive RF in a patient with B-cell lymphoproliferative disorder, which has not been previously reported from Pakistan. Hence, this case augments the need to do research on the association between B-cell lymphoproliferative disorder and IgG4-related RF.
